# Learning and altering behaviours by reinforcement: Neurocognitive differences between children and adults

**DOI:** 10.1016/j.dcn.2013.12.001

**Published:** 2013-12-07

**Authors:** E. Shephard, G.M. Jackson, M.J. Groom

**Affiliations:** Division of Psychiatry, University of Nottingham, Institute of Mental Health, University of Nottingham Innovation Park, Jubilee Campus, Triumph Road, Nottingham NG7 2TU, UK

**Keywords:** Development, Reinforcement learning, P3, Feedback-related negativity (FRN)

## Abstract

This study examined neurocognitive differences between children and adults in the ability to learn and adapt simple stimulus–response associations through feedback. Fourteen typically developing children (mean age = 10.2) and 15 healthy adults (mean age = 25.5) completed a simple task in which they learned to associate visually presented stimuli with manual responses based on performance feedback (acquisition phase), and then reversed and re-learned those associations following an unexpected change in reinforcement contingencies (reversal phase). Electrophysiological activity was recorded throughout task performance. We found no group differences in learning-related changes in performance (reaction time, accuracy) or in the amplitude of event-related potentials (ERPs) associated with stimulus processing (P3 ERP) or feedback processing (feedback-related negativity; FRN) during the acquisition phase. However, children's performance was significantly more disrupted by the reversal than adults and FRN amplitudes were significantly modulated by the reversal phase in children but not adults. These findings indicate that children have specific difficulties with reinforcement learning when acquired behaviours must be altered. This may be caused by the added demands on immature executive functioning, specifically response monitoring, created by the requirement to reverse the associations, or a developmental difference in the way in which children and adults approach reinforcement learning.

## Highlights

• Developmental differences in acquiring and adapting behaviours by reinforcement were examined.
• Children and adults acquired simple new behaviours by feedback comparably.
• Children's performance was more disrupted than adults’ when adapting behaviours.
• P3 ERP changes indicated children consolidated adapted behaviours less than adults.
• FRN ERP changes showed children relied more on feedback than adults in adaptation.

## 1 Introduction

### 1.1 Reinforcement learning in development

The ability to learn and modify behaviours based on the positive and negative outcomes of our actions is an important skill used throughout the lifespan. This skill, known as reinforcement learning (Holroyd and Coles, 2002, Thorndike and Bruce, 1911), may be particularly valuable in the first two decades of life, affording the naïve developing child an effective method of identifying advantageous behaviours and discerning when and how learned actions should be adapted for changing contexts. Indeed, impaired reinforcement learning has been implicated in the pathology of several neurodevelopmental disorders, including Tourette syndrome and ADHD (Marsh et al., 2004, Sagvolden et al., 2005), although the precise deficits in these conditions are unclear. A thorough understanding of the typical development of reinforcement learning may help clarify these deficits, but few studies have examined this aspect of cognitive development.

### 1.2 Differences in reinforcement learning across typical development

Previous studies have consistently reported performance differences between children and adults in reinforcement learning. Younger children are less accurate when learning associations between stimuli and responses (S–R associations) by positive and negative feedback than older children and adults (Baldwin et al., 2012, Crone et al., 2004). Children learn at a slower rate than adults (Crone et al., 2004) and show particular difficulties when reinforcements are inconsistent. Specifically, performance differences between children and adults increase when feedback is probabilistic and does not correctly reinforce performance 100% of the time (Eppinger et al., 2009, Hämmerer et al., 2010).

Neural processes underlying these developmental differences have been examined using EEG, particularly the feedback-related negativity (FRN) event-related potential (ERP). The FRN is a negative deflection in the waveform at ∼250 ms following feedback (Miltner et al., 1997). FRN amplitude is larger following negative than positive feedback, and in some studies positive feedback elicits a positive-going deflection in the FRN time-range, the feedback-positivity (FP) (Holroyd et al., 2008). Evidence suggests the FRN/FP is generated by prefrontal cortical regions associated with performance monitoring, and reflects the processing of dopaminergic reinforcement learning signals triggered by feedback indicating behaviour was better or worse than expected (Bellebaum and Daum, 2008, Luque et al., 2012, Oliveira et al., 2007). FRN/FP amplitudes decrease during a reinforcement learning episode, likely reflecting decreased reliance on external feedback with increasing knowledge of the to-be-learned behaviours (Eppinger et al., 2009, Holroyd and Coles, 2002).

Children show less enhancement of the FRN for negative compared with positive feedback, suggesting children are poorer at differentiating between types of feedback than adults (Hämmerer et al., 2010). The authors suggest this may explain why learning is more disrupted in children when feedback is probabilistic and difficult to discriminate. FP amplitude decreases less across learning in children than adults and ERP correlates of monitoring errors in performance differentiate less between correct and error responses in children than in adults (Eppinger et al., 2009). Based on these differences between children and adults, Eppinger et al. (2009) suggested that children have weaker internal representations of whether a response is correct or erroneous, resulting in a greater reliance on feedback processing to achieve successful performance. In a recent review of this literature, Hämmerer and Eppinger (2012) proposed that increasing reinforcement learning ability reflects developing efficiency in processing feedback, using reinforcements effectively to guide goal-directed behaviour, and building internal representations of correct behaviours, as prefrontal cortical regions mature.

However, due to the scarcity of research in this area further studies are needed (Hämmerer and Eppinger, 2012). Furthermore, previous research has not addressed an important aspect of reinforcement learning, that is, the ability to alter and re-learn behaviours following changes in reinforcements. A robust finding in the executive function literature is that children are poorer than adults in switching to new behaviours when prompted by cues (Koolschijn et al., 2011). This suggests that children will have particular difficulty with learning when reinforcement contingencies change. Furthermore, the learning tasks used previously have been complicated, with multiple feedback conditions presented for different S–R associations within task blocks, creating considerable working memory demands (Crone et al., 2004, Eppinger et al., 2009, Hämmerer et al., 2010). Crone et al. (2004) and Eppinger et al. (2009) controlled for this problem by allocating children extra response time, but nevertheless the difficulty of these tasks may have enhanced developmental differences.

### 1.3 The current study

The study aims were firstly to further investigate neurocognitive differences in the typical development of reinforcement learning using a simple task designed to reduce the influence of age-related performance differences on ERP correlates of learning. The intention was to ensure all participants could perform the task adequately regardless of age so that any ERP differences are more likely to reflect differences in the recruitment of neural networks underlying task performance, rather than floor or ceiling effects in one age group. Secondly, to assess developmental differences in the ability to change and re-learn acquired behaviour in response to altered reinforcement contingencies we compared children aged 9–11 years with adults aged 21 years and over. Our aim was to establish whether children differ from adults in behavioural and brain correlates of learning before they undergo the significant maturational changes that take place during adolescence. During EEG recording typically developing children and adults performed a task in which they learned four S–R associations by positive and negative feedback and then reversed the associations after an unexpected change in reinforcement contingencies. Changes in performance and feedback processing, indexed by the FRN, related to learning and reversal were examined across the task and between age groups. Additionally, changes in the P3 ERP, a positive deflection at ∼300 ms post-stimulus, were examined. P3 amplitude increases with progressing reinforcement learning in adults, which is thought to reflect increasing consolidation of to-be-learned behaviours (Rose et al., 2001). The P3 may further elucidate neurocognitive differences between children and adults, for example, children may show weaker consolidation of associations than adults reflected by smaller P3 amplitude increases with learning. We predicted children would show smaller learning-related changes in performance and ERP amplitudes during the initial acquisition of S–R mappings than adults, reflecting poorer learning ability at this age. Further, we expected children to show greater disruptions to performance and greater reliance on feedback, indexed by smaller FRN amplitude changes, when the reversal occurred.

## 2 Method

### 2.1 Participants

Fourteen 9–11 year olds (12 male, mean age: 10.2 years) and 15 adults (5 male, mean age: 25.5 years) were recruited from local primary schools and the University of Nottingham, UK to take part in this study. Participants were typically developing with no known neurological or psychiatric problems which may have affected brain function, right-handed (determined by the dominant hand for writing) and had normal or corrected-to-normal vision. Participants were tested in accordance with procedures approved by the University of Nottingham Medical School Ethics Committee and/or the East Midlands NHS Research Ethics Committee. Monetary reimbursement (£10) was provided for taking part.

### 2.2 Reinforcement learning task and testing procedure

**Fig. 1:**
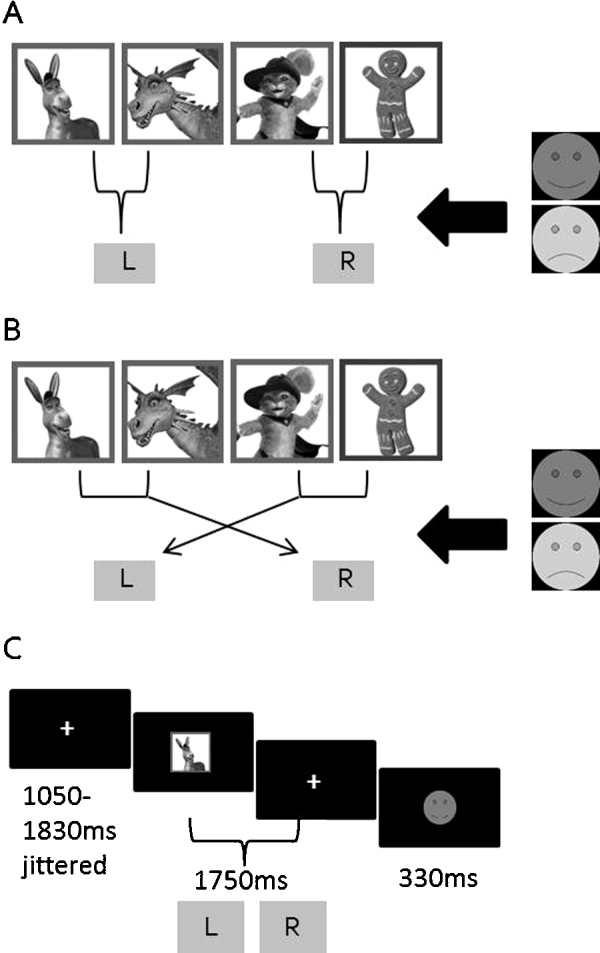
Task diagram. (A) Acquisition task period (blocks 1–3). Children learned which buttons (left/right) to press for each character stimulus. Two characters required right responses; two required left responses. Children began by guessing which button to press for each character. Feedback was provided to inform whether that response was correct (smiling face) or incorrect (sad face) for the character. Children were expected to remember (learn) which responses were correct for each character and produce those responses on all trials. Feedback was provided throughout. (B) Reversal task period (blocks 4–5). The correct responses for each character reversed unexpectedly and children had to re-acquire the correct S–R associations using feedback. For example, the two characters previously associated with a right response were negatively reinforced when this S–R association was produced, indicating the child must change their response to a left button press. Feedback was provided throughout. (C) Trial structure. Every trial began with a fixation screen. Next, one of the stimuli was presented followed by a second fixation screen, during which time the participant responded within a 1750 ms time limit. Every trial ended with a feedback display.

The reinforcement learning task (Fig. 1) required participants to learn by trial-and-error, using deterministic (always valid) performance feedback, to associate a set of two visual stimuli with a right hand button-press and another two stimuli with a left hand button-press. Three blocks of trials were presented for participants to learn the stimulus–response (S–R) associations. The S–R mappings reversed unexpectedly in a fourth block, requiring participants to re-learn the correct response for each stimulus. In a fifth block, the mappings remained reversed. Every block contained 48 trials, with each stimulus presented 12 times in random order in each block. Particular S–R associations were counterbalanced across participants. Stimuli were four cartoon characters from a popular animated film, presented in colour and surrounded by a rectangular 3 mm thick green frame. Stimuli measured 60 mm × 57 mm including the frame. Circular yellow happy-face images and blue sad-face images (both 60 mm in diameter) were used as positive and negative feedback. The words ‘Too slow!’ (10 mm × 90 mm) were displayed in green for late responses.

On each trial, a white fixation cross (7 mm × 7 mm) was presented for a jittered duration of 1050–1830 ms followed by one of the four stimuli for a maximum duration of 1475 ms. Stimulus presentation was terminated by the response and replaced by a second white fixation cross. Duration of the second fixation was dependent on the timing of the response, increasing with short latency responses and decreasing with long latency responses, resulting in a fixed time-window of 1750 ms between stimulus onset and fixation offset. Participants responded using the left/right buttons on a Cedrus RB-530 response button box (Cedrus Corporation, San Pedro, CA). Finally, feedback was displayed for 330 ms. Correct/incorrect feedback was displayed if the participant responded before fixation offset; ‘too slow’ feedback was displayed otherwise to encourage prompt responses. All task objects were centrally presented on a black background on a Viglen computer (43 cm monitor and 1024 × 768 pixels screen resolution). The task was programmed using E-Prime version 1.2 software (Psychology Software Tools Inc.).

After EEG set-up and task instructions, participants were seated in a dimly lit room at a distance of 60 cm from the monitor. Four practice trials (one per stimulus) were completed followed by the five task blocks separated by self-paced rest breaks. The task was to gain as many points as possible by learning the correct button-press for each stimulus. One point was awarded per correct response and the number of points won was displayed after each block. Participants were instructed to attend closely to the feedback to ensure they were aware of the change to response mappings but were not told when this would occur.

### 2.3 Electrophysiological recording and data processing

EEG was recorded continuously throughout task performance using a Biosemi Active II recording system (Biosemi, Amsterdam, The Netherlands) from 128 silver/silver chloride (Ag/AgCl) scalp electrodes placed according to the 5–20 system (Oostenveld and Praamstra, 2001). The data were referenced online to the Common Mode Sense (CMS) electrode located to the left of Cz on the scalp, and sampled at a rate of 512 Hz. Extra electrodes were placed on the inner orbital ridge and outer canthus of each eye and the right and left mastoids to record eye movements and non-ocular artefacts. Data were processed offline using Brain Vision Analyser 2.0 (Brain Products, Munich, Germany). Flat or noisy channels were removed prior to data processing. The data were re-referenced to the average of the scalp electrodes and filtered with 0.5 Hz high-pass, 30 Hz low-pass, notch 50 Hz zero-phase Butterworth 24 dB slope filters. Ocular artefacts were corrected using the Gratton and Coles regression method (Gratton et al., 1983). The data were segmented into learning blocks (1–5). Within these blocks stimulus- and feedback- locked epochs were created by segmenting the data in time from −200 ms to +1000 ms around stimulus/feedback onset respectively. Epochs were baseline-corrected using a pre-stimulus/pre-feedback reference period of −200 to 0 ms. Epochs were rejected if they contained amplitudes greater than ±90 μv. Epochs were averaged within each learning block to create separate stimulus-locked and feedback-locked ERPs for blocks 1–5. Correct trials (minimum of 20 trials) only were included in the average. No participants were excluded for failing to meet this criterion. However, the average number of trials included in the adults’ waveforms was significantly greater than the number included in the waveforms of children for each learning block (block 1: children mean 37, adult mean 42, *p* = .01; block 2: children mean 41, adult mean 45, *p* = .005; block 3: children mean 42, adult mean 45, *p* = .02; block 4: children 42, adult 46, *p* = .001; block 5: children 39, adult 45, *p* = .006).

### 2.4 Analysis methods

Behavioural performance was summarised in two ways. First, accuracy (% correct trials) and median RTs (for correct trials) were computed as an index of global performance in each block. Second, each block was divided into four quarters of 12 trials per quarter (quarter 1: first 12 trials in a block, quarter 2: second 12 trials, quarter 3: third 12 trials, quarter 4: last 12 trials). Each participant's accuracy (% correct trials) within each quarter was computed for each block. This was done to provide a measure of the extent to which learning of the S–R associations improved across trials within blocks and whether this within-block learning differed between children and adults.

Electrophysiological correlates of reinforcement learning were the stimulus-locked P3 and feedback-locked FRN ERP components. In early research the FRN was measured in incorrect trials, or computed as the difference in electrophysiological activity between correct and incorrect trials (Holroyd and Coles, 2002). However, recent research indicates that learning-related changes in the FRN are most prominent in correct trials, that is, following positive feedback (Arbel et al., 2013, Eppinger et al., 2009, Luque et al., 2012), and therefore the FRN was measured in correct trials only in this study. Based on parameters used in previous research and inspection of the grand and individual average waveforms, the stimulus-locked P3 was defined as the most positive peak in channel Pz in the time period 400–600 ms post-stimulus and the feedback-locked FRN was defined as the most negative peak in channel FCz in the 200–350 ms (adults) or 250–400 ms (children) post-feedback period. Peak amplitudes of the P3 and FRN were extracted for each learning block and used in analyses. Following previous authors examining learning-related differences in the FRN between children and adults (Eppinger et al., 2009), we also measured the FRN with respect to the preceding positive peak in the waveform (time-range 150–300 ms). Due to space constraints the results of this peak-to-peak analysis are reported only where they differ from those of the main peak analyses. The full set of data for the peak-to-peak analysis is available from the authors upon request.

To test the hypothesis that children show poorer learning of S–R associations than adults, mixed-model ANOVAs were performed on the data from the acquisition phase, namely task blocks 1–3. ANOVA models consisted of within-subjects factor block (3 levels) and between-subjects factor age (2 levels) and were run separately for each dependent variable (accuracy, RT, P3 amplitude, FRN amplitude). To test the hypothesis that children will experience greater disruption than adults when the associations change, mixed-model ANOVAs with within-subjects factor block (3 levels) and between-subjects factor age (2 levels) were conducted on the accuracy, RT, P3 and FRN data from the reversal phase of the task, that is, blocks 3–5. Greenhouse–Geisser corrections for violations of sphericity were used where appropriate. Significant main effects of block were further investigated with paired-samples *t*-tests to compare dependent variables across successive learning blocks (1–2, 2–3, 3–4, and 4–5). Significant interactions between block and age were further investigated by calculating difference scores to reflect the magnitude of change in a dependent variable (accuracy, RT, P3, FRN) in a given block compared with the previous block while taking into account group differences in initial performance and amplitude values. Difference scores were created for children and adults separately by subtracting dependent variable values in each block from those in the previous block, for example, RT in block 4 was subtracted from those in block 3 to characterise the extent to which RT decreased with the reversal of associations in block 4 compared with block 3. Independent-samples *t*-tests were used to compare difference scores across groups. To examine differences between children and adults in within-block learning improvements, mixed-model ANOVAs with one within-subjects factor of quarter (4 levels) and one between-subjects factor of age (2 levels) were conducted for each learning block separately. Significant main effects of quarter were further investigated using paired-samples *t*-tests to compare accuracy between successive quarters; significant interactions between quarter and age group were further investigated using independent-samples *t*-tests to compare accuracy within individual quarters between age groups. Finally, to determine whether ERP amplitudes related to task performance, Pearson correlation coefficients were computed between each of the performance and electrophysiological variables across learning blocks in children and adults separately.

## 3 Results

### 3.1 Behavioural reinforcement learning effects

#### 3.1.1 Acquisition phase (blocks 1–3)

##### 3.1.1.1 Accuracy

**Fig. 2:**
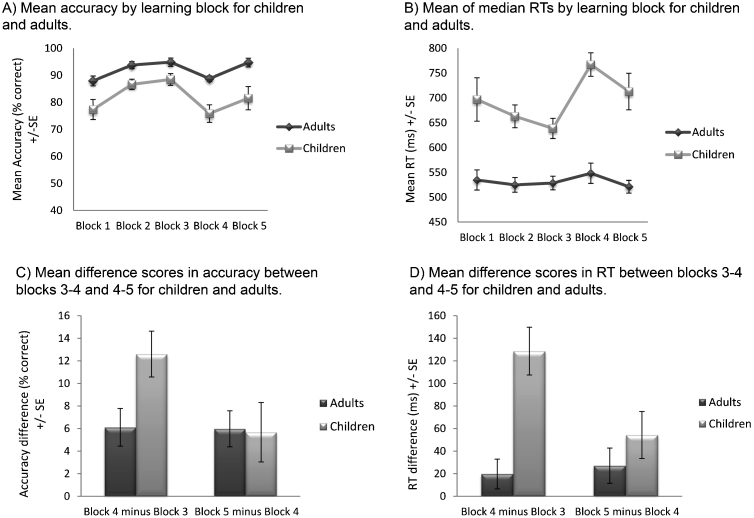
Performance data. Mean accuracy (A) and mean RT (B) data are shown plotted by learning block for children and adults. Mean difference scores in accuracy (C) and RT (D) between blocks 3–4 and 4–5 for children and adults. In all plots error bars represent standard error of the group mean.

Accuracy rates increased significantly across task blocks (*F* (2, 54) = 22.84, *p* < .001, *η*^2^ = .458) (Fig. 2) but this effect did not interact with age. Planned paired *t*-tests showed that, across groups, accuracy increased significantly from block 1 to 2 (*t* (28) = −4.34, *p* < .001 (1-tailed), *d* = −.76) but not from block 2 to 3 (*p* > .05). As predicted, children were less accurate than adults (*F* (1, 27) = 9.49, *p* = .005, *η*^2^ = .260) (Fig. 2).

##### 3.1.1.2 RT

There was no main effect of block or block × age interaction for RT, but children were significantly slower than adults overall (*F* (1, 27) = 21.01, *p* < .001, *η*^2^ = .438) (Fig. 2).

##### 3.1.1.3 Within-block learning

Analysis of within-block changes in accuracy across quarters in block 1 revealed a significant main effect of quarter (*F* (3, 81) = 9.07, *p* < .001, *η*^2^ = .25) but this did not interact with age (*p* > .1). Paired-samples *t*-tests revealed that accuracy increased significantly (across age groups) from the first to second quarter of block 1 (*t* (28) = −5.62, *p* < .001) but did not differ between remaining quarters (*p* > .1). Across all quarters, children were significantly less accurate than adults (*F* (1, 27) = 6.54, *p* = .02, *η*^2^ = .20). Similarly, across age groups accuracy differed significantly between quarters in block 2 (*F* (3, 81) = 3.67, *p* = .02, *η*^2^ = .12), which reflected increases in accuracy from the first to second quarter of the block (*t* (28) = -2.31, *p* = .03), the second to third quarters (*t* (28) = 2.08, *p* = .05) and the third to final quarters (*t* (28) = −2.24, *p* = .03). Again, children were significantly less accurate than adults across all quarters in block 2 (*F* (1, 27) = 1.67, *p* = .01, *η*^2^ = .22), but this did not interact with quarter (*p* > .1). In block 3, accuracy did not differ between quarters (*p* > .1) and there was no quarter by age group interaction (*p* > .1). Children were significantly less accurate than adults across all quarters in block 3 (*F* (1, 27) = 5.18, *p* = .03, *η*^2^ = .16). Plots of these data are available from the authors upon request.

#### 3.1.2 Reversal phase (blocks 3–5)

##### 3.1.2.1 Accuracy

Accuracy differed significantly across task blocks (accuracy: *F* (2, 54) = 19.68, *p* < .001, *η*^2^ = .422) and this effect interacted significantly with block (*F* (2, 54) = 3.23, *p* = .047, *η*^2^ = .107). Across groups, paired *t*-tests comparing successive blocks showed that accuracy significantly decreased from block 3 to 4 (*t* (28) = 6.49, *p* < .001 (1-tailed), *d* = .98) and increased from block 4 to 5 (*t* (28) = -3.91, *p* < .001 (1-tailed), *d* = .47). To investigate the interaction, independent samples *t*-tests were performed to compare the between block difference scores in children and adults, revealing a greater decrease in accuracy (*t* (27) = 2.49, *p* = .01 (1-tailed), *d* = −.89) from blocks 3 to 4 in children than in adults (Fig. 2). Children were significantly less accurate (*F* (1, 27) = 12.21, *p* = .002, *η*^2^ = .311) than adults overall.

##### 3.1.2.2 RT

RT differed significantly across task blocks: *F* (2, 54) = 15.02, *p* < .001, *η*^2^ = .357 and there was a significant block × age group interaction (*F* (2, 54) = 8.75, *p* = .001, *η*^2^ = .245). Planned paired *t*-tests showed RT significantly increased from block 3 to 4 (*t* (28) = −4.56, *p* < .001 (1-tailed), *d* = −.63) and decreased from block 4 to 5 (*t* (28) = 3.11, *p* = .002 (1-tailed), *d* = −.29) across groups. Analysis of difference scores between blocks showed there was a greater increase in RT (*t* (27) = 4.37, *p* < .001 (1-tailed), *d* = −1.63) from blocks 3 to 4 in children than in adults (Fig. 5) but no group difference in RT decreases across blocks 4–5. Children were significantly slower than adults overall (*F* (1, 27) = 39.71, *p* < .001, *η*^2^ = .595)

##### 3.1.2.3 Within-block learning

In block 4, accuracy differed significantly between quarters (*F* (3, 81) = 8.11, *p* < .001, *η*^2^ = .23) but this did not interact with age group (*p* > .1). Across age groups, accuracy improved significantly from the first to second quarter of trials (*t* (28) = −2.86, *p* = .008) but did not differ between the remaining quarters (*p* > .1). Across all quarters, children were significantly less accurate than adults in block 4 (*F* (1, 27) = 14.96, *p* = .001, *η*^2^ = .56). In block 5, accuracy did not differ between quarters (*p* > .1) and there was no interaction between quarter and age (*p* > .1). Children were significantly less accurate than adults across all quarters (*F* (1, 27) = 6.28, *p* = .02, *η*^2^ = .19). These data are available from the authors upon request.

### 3.2 Electrophysiological reinforcement learning effects

#### 3.2.1 Acquisition phase (blocks 1–3)

##### 3.2.1.1 P3

**Fig. 3:**
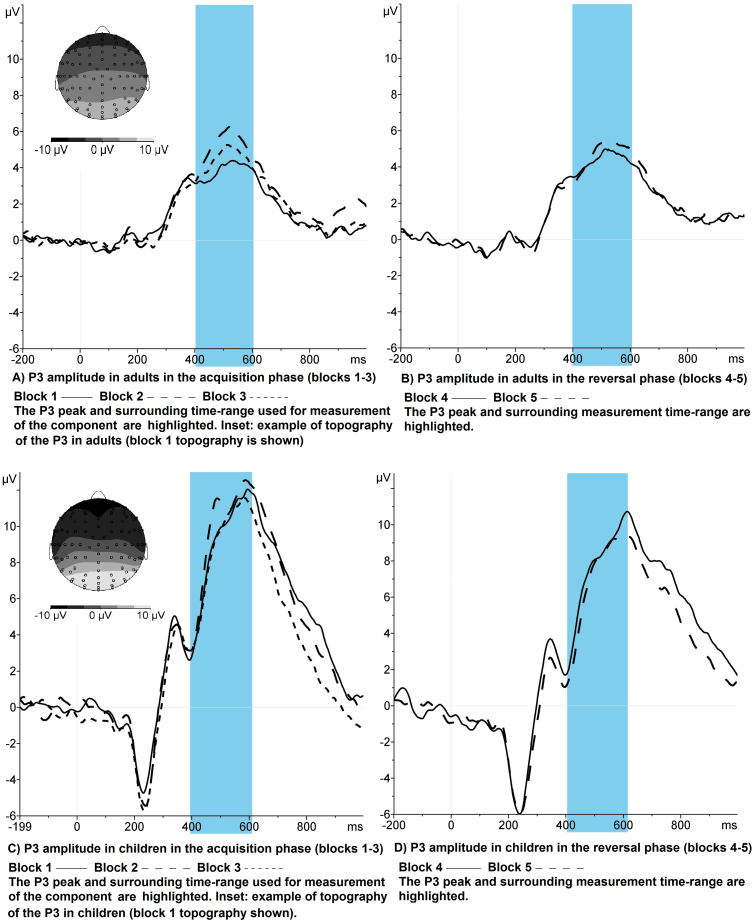
P3 amplitude by learning block in children and adults. The plots in panels A and B show stimulus-locked waveforms at electrode Pz for the acquisition and reversal phases of the task respectively, in the adult group. Panels C and D display stimulus-locked waveforms at Pz for the acquisition and reversal phases respectively in the child group. Time is shown on the *x*-axis in milliseconds, with 0 representing stimulus onset and amplitude on the *y*-axis in microvolts. The P3 peak and the time-range in which the P3 was measured (400–600 ms) are highlighted in each plot. The topographic maps shown in the insets of panels A and C represent the scalp activity in microvolts in greyscale during the P3 time-range in block 1 for adults and children respectively. These topographical plots are provided to illustrate that the P3 showed the typical topography for this component in the adult and child groups.

Amplitudes were significantly greater in children than adults (*F* (1, 27) = 14.48, *p* = .001, *η*^2^ = .349) and differed significantly by task block (*F* (2, 54) = 3.51, *p* = .04, *η*^2^ = .115) but there was no interaction between block and age (Fig. 3). Across groups, P3 amplitude increased significantly from block 1 to 2 (*t* (28) = −2.59, *p* = .07 (1-tailed), *d* = −.21) and decreased significantly from block 2 to 3 (*t* (28) = 2.51, *p* = .009, *d* = .17).

##### 3.2.1.2 FRN

**Fig. 4:**
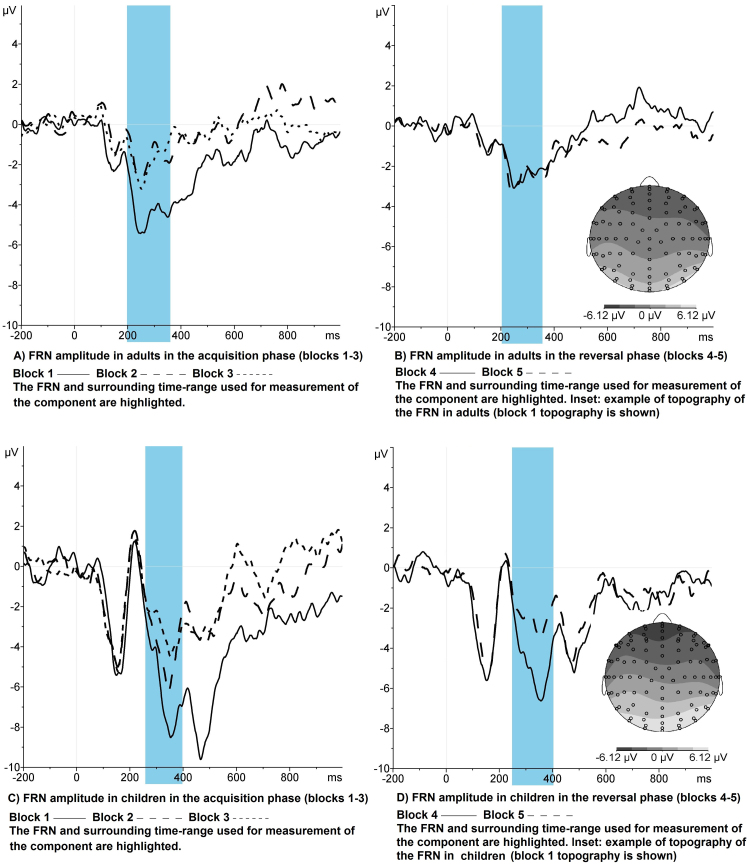
FRN amplitude by learning block in children and adults. The plots in panels A and B show feedback-locked waveforms at electrode FCz for the acquisition and reversal phases of the task respectively, in the adult group. Panels C and D display feedback-locked waveforms for the acquisition and reversal phases respectively in the child group. Time is shown on the *x*-axis in milliseconds, with 0 representing stimulus onset and amplitude on the *y*-axis in microvolts. The FRN peak and the time-range in which the FRN was measured are highlighted in each plot. The topographic maps shown in the insets of panels B and D represent the scalp activity in microvolts in greyscale during the FRN time-range in block 1 for adults and children respectively. These topographical plots are provided to illustrate that the FRN showed the typical topography for this component in both age groups.

Children's FRN amplitudes were significantly larger than those of adults (*F* (1, 27) = 6.54, *p* = .02, *η*^2^ = .195). FRN amplitude decreased significantly across blocks (*F* (2, 54) = 18.63, *p* < .001, *η*^2^ = .408) but this effect did not interact with age (Fig. 4). FRN amplitude decreased significantly from block 1 to 2 (*t* (28) = −4.85, *p* < .001 (1-tailed), *d* = −.89) but not from block 2 to 3 (*p* > .1) across groups.

#### 3.2.2 Reversal phase (blocks 3–5)

##### 3.2.2.1 P3

**Fig. 5:**
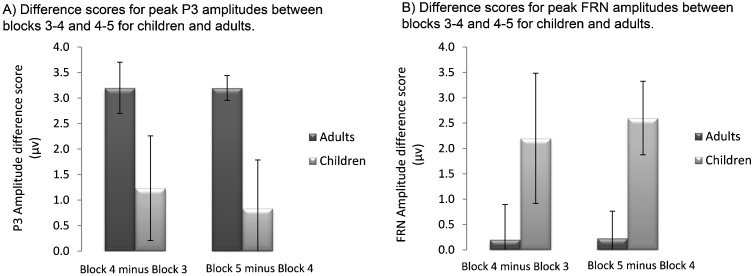
Difference scores for peak P3 amplitudes (A) and peak FRN amplitudes (B) between blocks 3–4 and 4–5 for children and adults. Error bars represent standard error of the group mean.

P3 amplitudes changed significantly across blocks (*F* (2, 54) = 8.31, *p* = .001, *η*^2^ = .235) and, as predicted there was a significant block × age interaction (*F* (2, 54) = 6.97, *p* = .002, *η*^2^ = .205). Across groups, P3 amplitude decreased significantly from block 3 to 4 (*t* (28) = 3.74, *p* < .001 (1-tailed), *d* = .40) and increased significantly from block 4 to 5 (*t* (28) = −1.88, *p* = .04, *d* = −.25). Analysis of difference scores to explore the block × age interaction showed that the amplitudes of adults decreased significantly more from blocks 3 to 4 (*t* (27) = −3.80, *p* < .001 (1-tailed), *d* = .63) and increased significantly more from blocks 4 to 5 (*t* (27) = 2.13, *p* = .02, *d* = .80) than those of children (Fig. 5). Amplitudes were overall significantly larger in children than adults (*F* (1, 27) = 20.96, *p* < .001, *η*^2^ = .437) (Fig. 3).

##### 3.2.2.2 FRN

FRN amplitudes were significantly larger in children than adults (*F* (1, 27) = 5.44, *p* = .03, *η*^2^ = .168) and differed at a trend level between blocks (*F* (1.59, 43) = 3.43, *p* = .07, *η*^2^ = .101). There was also a significant interaction between block and age (*F* (1.59, 43) = 3.43, *p* = .05, *η*^2^ = .113). The difference in amplitude between blocks 4 and 5, but not between blocks 3 and 4, was significantly greater in children than adults (*t* (27) = 3.44, *p* < .001 (1-tailed), *d* = −1.06). There was no effect of age group on the difference in amplitude between blocks 3 and 4 (Fig. 5). The trend-level interaction between block and age did not remain when peak-to-peak measures of the FRN were analysed (*p* = .18); however, examination of the difference in FRN amplitude between blocks 4 and 5 remained significantly larger in children than adults.

### 3.3 Relationships between performance and electrophysiological variables

**Fig. 6:**
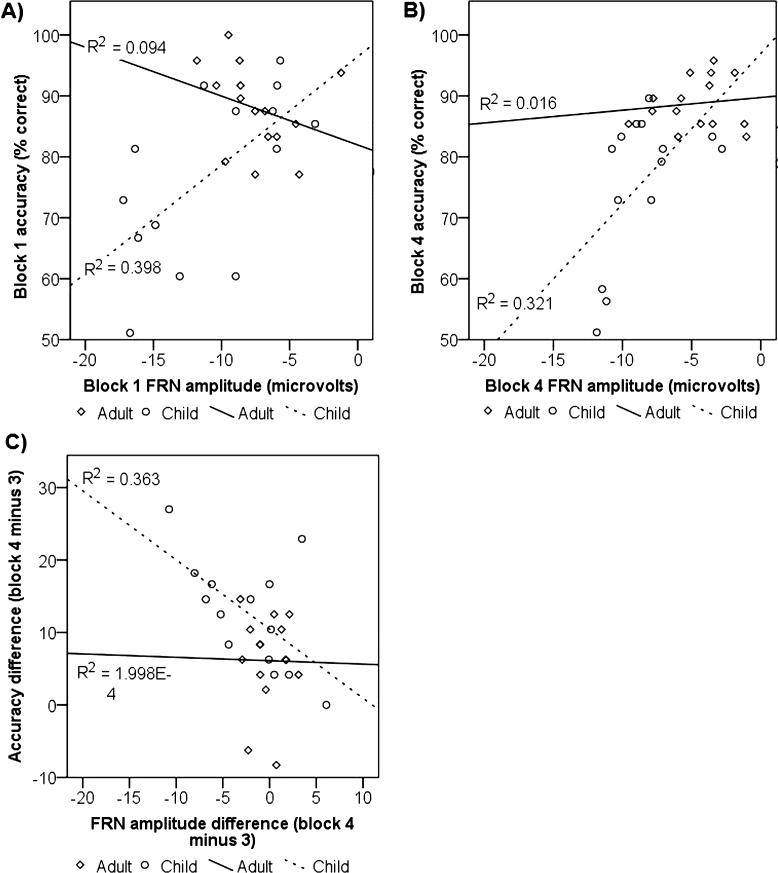
Scatterplots showing correlations between FRN amplitude and accuracy. The plots show the correlation between FRN amplitude and accuracy in block 1 (A) and block 4 (B), and between the difference scores characterising the degree of change in FRN amplitude and accuracy in blocks 3–4 (C) separately for children and adults. The correlations were significant in children only.

To investigate whether ERP amplitudes were related to task performance, Pearson correlation coefficients were computed between each of the performance (accuracy, RT in each block; difference scores for accuracy and RT change across blocks) and electrophysiological variables (P3 and FRN amplitude in each block; difference scores for P3 and FRN across blocks) in children and adults separately. In children only, accuracy and FRN amplitude were significantly positively correlated in block 1 (*r* (14) = .631, *p* = .02, *r*^2^ = .40) and block 4 (*r* (14) = .566, *p* = .04, *r*^2^ = .32), reflecting more positive, i.e. reduced, FRN amplitude in participants with higher accuracy levels in the first block of the acquisition phase and on reversal of mappings in block 4 (Fig. 6). Furthermore, the extent to which FRN amplitude increased from block 3 to 4 was significantly negatively associated with increases in accuracy from block 3 to 4 in children (*r* (14) = −.603, *p* = .02, *r*^2^ = .36). No other correlations reached significance in children or adults.

## 4 Discussion

This study investigated neurocognitive differences in reinforcement learning in typically developing children and adults. The aims were to extend previous research by examining developmental differences when task difficulty was appropriate for children and when unanticipated changes in response contingencies were introduced. Analysis of performance and electrophysiological activity during a simple reinforcement learning and reversal task in children and adults revealed two important findings. First, contrary to our predictions, children and adults did not differ in learning-related performance or ERP changes during the initial acquisition of S–R associations. Second, in support of our prediction, performance was significantly more disrupted in children than adults when reversal of S–R associations was required, and this was accompanied by developmental differences in neural correlates of consolidation and feedback processing, the P3 and FRN event-related potentials. These findings are discussed below.

### 4.1 Acquisition of simple new behaviours by reinforcement

Children and adults showed equivalent increases in accuracy and P3 amplitude and decreases in FRN amplitude as they learned the S–R associations. Therefore, in contrast to previous research (Crone et al., 2004, Eppinger et al., 2009, Hämmerer et al., 2010) children in this study acquired and consolidated new behaviours and gradually decreased their use of external feedback at the same rate as adults. Accuracy significantly correlated with FRN amplitude during the first task block in children, indicating that feedback processing was related to the correct production of S–R associations in children in this study. This extends previous research by indicating that feedback processing and guidance of goal-directed behaviour by reinforcement information is not deficient in children compared with adults, as has previously been proposed (Hämmerer and Eppinger, 2012). Our findings indicate that when reinforcement learning is non-probabilistic the neural mechanisms underlying this basic form of learning work as efficiently in children as in adults. Problems with acquiring new behaviours may only appear in children when reinforcement learning becomes more complicated, for instance when reinforcements are unclear, for example probabilistic, and demands on other maturing cognitive functions such as working memory or executive function are high. As such, our findings highlight the importance of ensuring task difficulty is appropriate for children in developmental investigations of reinforcement learning.

### 4.2 Developmental differences in altering learned behaviours by reinforcement

Performance was significantly more impaired in children than adults when reinforcements changed and the reversal of S–R associations was required in block 4 of the task. Nevertheless, following the reversal children improved their performance at the same rate as adults (task block 5). These findings suggest that children have specific performance difficulties when unexpected changes in reinforcements occur, but are eventually able to re-acquire simple behaviours in a similar manner to adults. Analysis of the P3 and FRN revealed further developmental differences in neurocognitive processes underlying performance.

The magnitude of P3 amplitude changes during learning can be considered to index the strength of internal representation of correct S–R associations in working memory (Barceló et al., 2000, Rose et al., 2001). P3 amplitude changes were significantly greater in adults than children, decreasing more during reversal of associations and increasing more with re-acquisition of reversed mappings, indicating that internal representations of the S–R associations underwent less adaptation and re-consolidation in children than adults. In contrast, FRN amplitude changes were greatest in children, decreasing more with re-learning of the associations in block 5 than in adults. Indeed, FRN amplitude showed little variation after the first task block in adults while a prominent increase with reversal and decrease with re-acquisition was observed in children, indicating that feedback processing varied more with reversal and re-learning in children than adults. Previous authors have emphasised that difficulties with feedback processing, resulting from immature performance monitoring functions of the developing prefrontal cortex, underlie children's poorer reinforcement learning performance (Hämmerer and Eppinger, 2012, Hämmerer et al., 2010). It has been suggested that children are less successful than adults in integrating feedback information with motor action plans, or that children use feedback in a less goal-directed manner than adults (Hämmerer and Eppinger, 2012, Hämmerer et al., 2010). In contrast to the latter proposal, our findings suggest that children do use feedback to drive goal-directed learning behaviour. Changes in FRN amplitude were associated with changes in performance accuracy in children when most re-learning was occurring (block 4). Furthermore, FRN changes were largest in children, indicating children were using feedback more than adults to guide behaviour. However, as children performed more poorly than adults, children may have had greater difficulty in integrating feedback information to consolidate S–R associations and so produce the correct behaviours, consistent with other work using a probabilistic learning task (Van Duijvenvoorde et al., 2013).

Errors were not sufficiently numerous to allow analysis of the ERN in this study. However, the profile of P3 and FRN effects here are similar to the ERN and FP findings reported by Eppinger et al. (2009), and support the proposal put forward by those authors that children build weaker internal representations of to-be-learned behaviours and engage in greater processing of external feedback than adults when alterations in reinforcement learning are required. This may be due to interference arising from the extra cognitive processing demands of reversing the S–R associations, such as the requirement to suppress the previously correct behaviours and produce new responses that conflict with the original S–R associations. A wealth of evidence demonstrates that such executive functions are poorer in children than adults (Johnstone et al., 2005, Ladouceur et al., 2007, Rueda et al., 2004). Therefore, it may be that these additional processing requirements reduce children's cognitive capacity for learning, decreasing the efficiency of the processes of consolidating the reversed S–R associations and integrating new feedback information with behaviour plans. Children may exercise greater feedback processing to compensate for these difficulties. Alternatively, the enhanced FRN in children may reflect a greater affective or motivational response to correct responses during the more challenging phases of the task. Amplitude of the FRN to negative feedback has been related to individual differences in punishment sensitivity in adolescence and adults (Santesso et al., 2011) and may reflect evaluation of good versus bad outcomes based on motivational as well as cognitive goals (Hajcak et al., 2006). It is possible therefore that the children in the present study invoked this evaluative process more strongly than adults having encountered greater difficulty during the reversal phase of the task. However, the present task was not designed with this question in mind and further research is needed to investigate the role of the FRN in children in this age range.

Another possible explanation for our findings is that children learn in a different manner from adults. Research in adults has shown that providing information about reward likelihood enhances the reinforcement learning process. For example, Li et al. (2011) and Walsh and Anderson (2011) compared adults’ performance on a probabilistic S–R learning task when no information about reinforcement probabilities was given and adults were required to learn the S–R associations solely by feedback, with a separate condition in which participants were instructed as to the probability that each S–R pair would be followed by valid feedback, for example that one S–R association would be correctly reinforced on 30% of trials. Adults’ performance increased gradually in the no-instruction learning condition, but began and remained at asymptote in the instruction condition. The enhancing effect of instruction on learning is suggested to reflect the top-down influence of rules for learning represented in prefrontal regions on striatal reinforcement learning mechanisms (Li et al., 2011).

In the current study, a rule for how the S–R associations should be re-learned would have been acquired easily after only a few trials in block 4 based on knowledge of what the original S–R mappings were and identifying that the mappings simply had to be reversed. If implemented, this rule would facilitate faster re-learning of the associations. Adults verbally reported that they realised the S–R combinations in block 4 were simply the opposite of those in blocks 1–3. Adults’ rapid increase in consolidation of the new S–R associations, improvement in performance and minimal variation of the FRN suggests that they used this inferred rule to guide re-learning rather than relied on external feedback. Children's slower consolidation of reversed S–R associations, more disrupted performance, and greater feedback processing suggests that they were relying on external reinforcement information rather than the internally derived rule for re-learning that adults appeared to employ. Therefore, a possible explanation for the developmental difference in performance and neurocognitive processing in the reversal phase is that unlike adults, children do not infer and use rules for learning, and instead rely on slower feedback-based learning. It is unclear whether this reflects an inability of children to infer learning rules and use them to drive performance due to under-developed prefrontal regions, or a strategic preference for experience-based learning in children. Future studies comparing instruction-based and experience-based learning in children and adults would be useful in clarifying this issue.

One final observation to discuss is the prolonged negativity following the FRN observed in the feedback-locked waveforms in all learning blocks in children but not in adults (Fig. 4). A detailed analysis of this component was beyond the scope of this article, but would be worthy of future research. It is likely that this second negative peak in the children reflects a second oscillation of the same on-going physiological process (feedback-processing), and may occur due to additional or more effortful processing of the feedback information in children to compensate for their greater difficulty in learning the S–R associations. Alternatively, this negativity might index different learning strategies used in children compared with adults. A recent study comparing feedback-locked potentials between groups of adults using different learning strategies to acquire new behaviours reported strategy-related differences in the morphology of positive feedback components (Rustemeier et al., 2013).

### 4.3 General developmental differences in performance and ERP amplitudes

In addition to learning-related developmental differences, children showed less accurate and slower performance and larger P3 and FRN amplitudes than adults overall. This is consistent with evidence that children's accuracy rates are lower and response times are slower than adults’ across a broad range of cognitive tasks, including executive function and attention (Burgund et al., 2006, Johnstone et al., 2005, Ladouceur et al., 2007). These differences are therefore more likely to be general indicators of proficiency in performing cognitive tasks requiring coordinated manual responses and are not specific to learning. The findings that children did not differ from adults in the degree to which accuracy improved within learning blocks further suggests that children were learning at the same rate as adults, and that accuracy differences reflected general performance differences rather than learning-related differences. However, it would be useful to investigate within-block changes in learning performance further in future research, perhaps by fitting curvilinear or exponential learning-slope functions, to more conclusively demonstrate that the rate at which children learned was comparable to that in adults. The present findings are consistent with previous reinforcement learning studies which have shown greater FRN amplitude in children than adults, possibly reflecting greater sensitivity to feedback in childhood than adulthood (Eppinger et al., 2009, Hämmerer et al., 2010). Other factors such as age differences in skull density, brain size and cortical folding cannot be ruled out (Segalowitz and Davies, 2004), although the finding reported here of greater learning effects on FRN amplitude in children than adults strengthens the hypothesis that the overall amplitude differences may reflect true differences in the electrical activity of neural networks supporting feedback processing. It must be noted here that the lower number of trials included in children's than adults’ waveforms may have resulted in differences in signal-to-noise ratio of the waveforms between age groups. In turn, this may have influenced the group differences in ERP components and caution should be exercised when interpreting the age-related ERP differences. On the other hand, the findings that trial number differences were present in all learning blocks but the age-related differences were restricted to particular blocks (3–5 for the P3 and 4–5 for the FRN) suggests that the group differences in learning-related changes in these ERP components were not solely due to signal-to-noise ratio differences.

## 5 Conclusions

The current findings revealed that children can perform as well as adults in acquiring simple new stimulus–response behaviours by reinforcement, providing the learning situation is uncomplicated with minimal demands on other cognitive abilities such as executive function and working memory. Moreover, neurocognitive processes of consolidating internal representations of correct behaviours and processing reinforcing feedback information are comparable between children and adults in simple learning situations. However, when modification of learned behaviours by reinforcement is required, children's performance is significantly more disrupted than that of adults, children show less consolidation of the new behaviours and greater reliance on feedback information than adults. These neurocognitive differences specific to altering reinforcement learning may reflect a different style of learning in children and adults, that is, internally inferred rule-based learning in adults compared with externally driven experience-based learning in children. Alternatively, children may experience a general reduction in the efficacy of reinforcement learning processes due to enhanced demands on executive function resulting from the requirement to modify behaviours.

## Conflicts of interest

The authors have no conflicts of interest.
